# The role of olfactory cues in mother–pup, groupmate, and sex recognition of lesser flat‐headed bats, *Tylonycteris pachypus*

**DOI:** 10.1002/ece3.8249

**Published:** 2021-10-30

**Authors:** Jie Liang, Jian Yang, Yi Chen, Libiao Zhang

**Affiliations:** ^1^ Guangdong Key Laboratory of Animal Conservation and Resource Utilisation Guangdong Public Laboratory of Wild Animal Conservation and Utilisation Institute of Zoology Guangdong Academy of Sciences Guangzhou China

**Keywords:** bats, groupmate recognition, mother‐pup recognition, scent, sex recognition, *Tylonycteris pachypus*

## Abstract

*Tylonycteris pachypus* is a gregarious bat species with tens of individuals in a colony. The mechanisms by which mother bats recognize their pups and adult bats recognize each other are not clear. We hypothesized that such recognition is achieved by chemical discrimination and performed experiments to test the hypothesis. Results showed that mother bats were much more attracted to the scent from their own pups. For adult bats, females were attracted to the scent from both male and female groupmates but had a higher preference to the scent from female than from male groupmates. Male bats were much more attracted to the scent from male groupmates while showed no preference to the scent from female groupmates. Within a group, both female and male bats had no difference in preference to the scent from the same or opposite sex. These results suggest that mother–pup and groupmate recognition of *T*. *pachypus* can be achieved by olfactory cues.

## Graphical abstract

We did three experiments with a Y‐maze, trying to know the role of odor in *Tylonycteris pachypus* daily communication. The results show that odorant choice is important in bats' social contacts and it works in different ways.

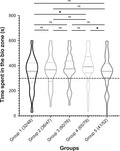

## 1 INTRODUCTION

The lesser flat‐headed bats, *Tylonycteris pachypus* (Chiroptera:Vespertilionidae), are one of the smallest mammals in the world (Zhang et al., 2007) and are distributed across Southeast Asia (Simmons, 2005). These bats roost in internode cavities of bamboo stems and normally form small groups (Medway & Marshall, 1972). The size of each group is in the range of 2–27 bats. Although a group normally consists of several females with one or multiple males, all‐male and all‐female groups and groups with solitary males have been observed (Hua et al., 2011; Medwaym & Marshall, 1970). Female bats typically produce twins (Medway & Marshall, 1972).

As vision is ineffective in complete darkness for most nocturnal mammals such as lesser flat‐headed bats that live in bamboo tubes (Chaverri et al., 2018), they use other means for recognition and communication. For instance, nursing *T*. *pachypus* bats leave their pups behind in their roost when they fly out to search for food (Medway, 1972). Upon return, they can unmistakingly find their own pups from a crowd of pups of various mother bats. A previous study showed that locational memory is involved in female‐pup reunions in bats (Mccracken, 1993). However, spatial memory is ineffective in a confined space of *T*. *pachypus* roost. As *T*. *pachypus* bats form stable associations within closed maternity colonies, females are highly philopatric (Zhang et al., 2004) in the recognition of their roostmates.

Bats have evolved a wide range of sensory mechanisms; some of which, such as vocalization and olfaction, are highly specialized (Altringham & Fenton, 2003). Vocalization allows bats to navigate by means of echolocation and to communicate and socialize with others. It is also used to express territoriality, courtship, aggression, isolation, and other social activities (Gould, 1971; Heckel & Helversen, 2003; Kanwal et al., 1994). Olfaction is used by bats for closed‐up recognition (Bradbury, 1977; Fenton, 1985; Mccracken, 1993), and profile scent is used for identification of other individuals (Bloss, 1999; Brooke & Decker, 1996). Scent may be created by a combination of glandular secretions, urine, feces, and bacterial communities (Nielson et al., 2006).

It has been shown that chemical signals are important media for age‐related recognition, mate choice, and mother–pup reunion in mammals (Lévy et al., 2004; Martín et al., 2014; Parrott et al., 2007). In this study, we hypothesized that *T*. *pachypus* bats can also achieve mother–pup and groupmate recognition and sex recognition by scent. To test our hypothesis, we performed mother–pup odorant choice (M‐POC), groupmate odorant choice (GOC), and sex odorant choice (SOC) experiments using a Y‐maze‐like arena. Results showed that *T*. *pachypus* bats can discriminate scent from their pups and groupmates. They can also discriminate sex by scent. In addition, female bats were found to be more attracted to scent from female than male groupmates.

## 2 METHODS

### 2.1 Bat collection

**FIGURE 1:**
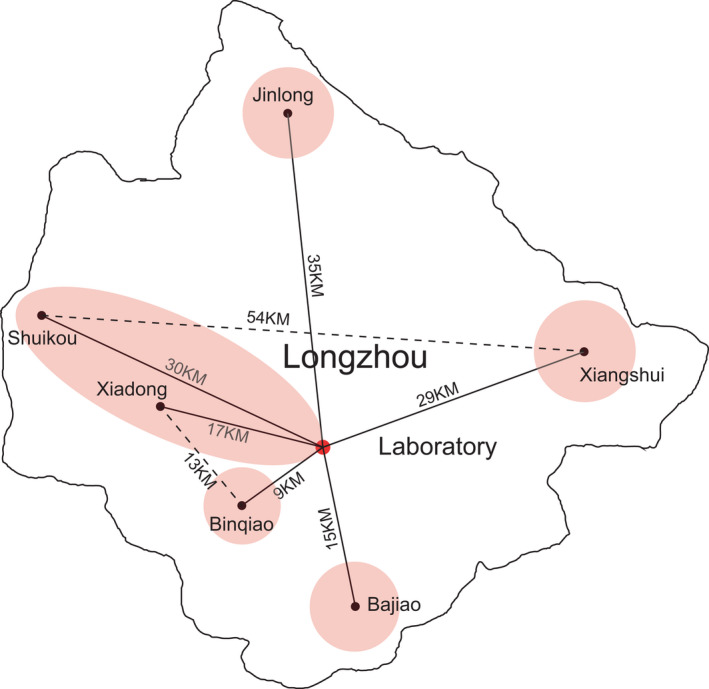
Sampling sites in Longzhou County, China

Bats used in this study were collected from 6 sites, including Jinlong, Shuikou, Xiadong, Binqiao, Bajiao, Xiangshui in Longzhou, China, from May to September 2011 (Figure 1). The farthest distance between two sampling sites, Shuikou and Xiangshui, is 54 km, and the shortest distance is 13 km (Figure 1) between Xiadong and Binqiao. Fission–fusion in which some bats switched among roosts was observed in some colonies, but bats from different colonies rarely visited each other as described previously (Zhang et al., 2004). Captured bats were transported to the laboratory in clear 25 cm (height) by 15 cm (width) thin cloth bags. Forearm length of each bat was measured using a vernier caliper (G13940, Guilin Measuring & Cutting Tools Works Co., Ltd., Guilin, China), which has an accuracy of 0.01 mm. Each bat was used only once in experiments. All bats were released back to their roosts the next day after the experiments were completed.

### 2.2 Experimental device

**FIGURE 2:**
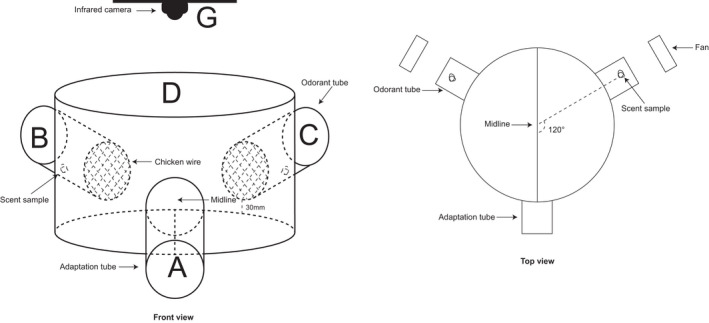
Maze apparatus used in experiments. A. Adaption tube; B and C. Odorant tubes; D. Midline. G. infrared camera. The angle between the adaption tube and each odorant tube is 120°

A Y‐shaped maze arena was used for all experiments (Figure 2). The central part of the device is a plastic bucket (diameter: 66.0 cm, height: 20.5 cm) with a transparent glass cover on top and is connected to 3 polyethylene tubes (diameter: 60 mm, length 100 mm, 30 mm above the floor of the bucket) (labeled A, B, and C in Figure 2). The outer end of B and C tubes, designated as odorant tubes, is open, and the other end, which links the tube to the arena, is covered with chicken wire. A scent sample to be tested is placed near the outer third of an odorant tube. The third tube is used for habituation and is designated as the adaptation tube (labeled A in Figure 2); both ends of this tube are covered with a door. An infrared camera is mounted 1 m above the arena (G) (Figure 2).

A sterile cotton ball was used to collect scent from various places of the body of a bat including anus, genitals, mouth, and nose by lightly wiping each area 10 times. Each scent‐impregnated sample was used immediately by placing it in an odorant tube for testing.

The bat to be tested was placed in the adaption tube to habituate for at least 5 min. The door to the arena was then open to allow the bat to crawl into the arena. If the bat failed to enter the arena within 3 min, the test was aborted. A tiny fan was used to blow the scent from the odorant tube to the arena for 1 min. The arena was divided along the midline into two zones. The amount of time within the 10‐min observation period the bat moved from the adaptation tube, passed the midline of the arena, and wandered in the zone near an odorant box was recorded using a PICO2000 multimedia digital video monitor connected to a computer. The device was washed with tap water thoroughly and wiped with 75% ethyl alcohol between tests.

### 2.3 Mother–pup odorant choice (M‐POC) experiment

A total of 302 female *T*. *pachypus* bats were tested in this experiment. The pups were divided into 5 age groups according to their forearm length as it is the most accurate and reliable index for age determination (Krochmal & Sparks, 2007; Kunz & Anthony, 1982). The average forearm lengths of the 5 groups are as follows: Group 1, ≤10 mm; Group 2, 10 mm‐≤15 mm; Group 3, 15 mm‐≤20 mm; Group 4, 20 mm‐≤25 mm; and Group 5, ≥25 mm (Table 1). Each experiment consisted of a female bat and two pup scent samples with one sample from a biological pup and the other from an alien pup. Biological pups were collected from a nursing mother bat while feeding. Alien pups were those from a different mother bat. The scent‐impregnated samples from a biological pup and an alien pup were placed separately in the two odorant tubes of the device for testing. Each scent‐impregnated cotton ball was used only once, and the two odorant tubes were used alternatively and randomly for biological and alien samples.

**TABLE 1:** Forearm length and body weight of pups in the mother–pup odorant choice experiment

Group	1 (*n* = 48)	2 (*n* = 47)	3 (*n* = 76)	4 (*n* = 79)	5 (*n* = 52)
Forearm length (mm)	9.53 ± 0.17	12.43 ± 1.60	17.72 ± 1.13	23.55 ± 1.06	26.11 ± 0.66
Body weight (g)	0.57 ± 0.10	1.31 ± 0.39	2.39 ± 0.32	2.95 ± 0.26	3.20 ± 0.23

The arena of the testing device was divided equally along the midline into two zones. The zone near the tube containing the scent sample from a biological pup was designated as the biozone, and the other near the tube containing the scent sample from an alien pup was designated as the alien zone. A mother bat was released from the adaptation tube to the arena. As the arena does not have sufficient height for the bat to fly, it crawled randomly and back and forth between the two zones but would spend more time in the zone near the tube containing the scent sample to which it was attracted to. The total amount of time that the bat spent in the biozone and the alien zone was recorded.

### 2.4 Groupmate odorant choice (GOC) experiment

A total of 243 bats (149 females and 94 males) were tested in this experiment. They were divided into 4 groups as follows: Group ♀|♀♀, a female bat with scent samples from a female groupmate and a female nongroupmate; Group ♀|♂♂, a female bat with scent samples from a male groupmate and a male nongroupmate; Group ♂|♀♀, a male bat with scent samples from a female groupmate and a female nongroupmate; and Group ♂|♂♂, a male bat with scent samples from a male groupmate and a male nongroupmate.

As in the M‐POC experiment, the arena was divided into two zones. The zone near the tube containing the scent sample from a groupmate was designated as the groupmate zone, and the other near the tube containing the scent sample from a nongroupmate was designated as nongroupmate zone. A bat was released into the arena, and the time it spent in the groupmate zone and the nongroupmate zone was recorded.

### 2.5 Sex odorant choice (SOC) experiment

A total of 91 bats (57 females and 34 males) were tested in this experiment. They were divided into two groups according to their sex as follows: Group ♀|♀♂, a female bat with scent samples from a female bat and a male bat of the same group; and Group ♂|♀♂, a male bat with scent samples from a male bat and a female bat of the same group.

As in M‐POC and GOC experiments, the arena was divided into two zones. The zone near the tube containing the scent sample from a female bat was designated as the female zone, and the other near the tube containing the scent sample from a male bat was designated as the male zone. A bat was released into the arena, and the time it spent in the female zone and the male zone was recorded.

### 2.6 Statistical method

The binary test was performed to determine which scent was more attractive to bats in all 3 experiments (M‐POC, GOC, and SOC). The Wilcoxon test was performed to determine the difference in scent preference among various groups of bats in M‐POC and GOC experiments, but not in the SOC experiment as it had only two groups of bats. These statistical analyses were carried out using the software SPSS, version 26 (IBM Corp. Released, 2019).

## 3 RESULTS

### 3.1 Mother–pup odorant choice (M‐POC) experiment

**FIGURE 3:**
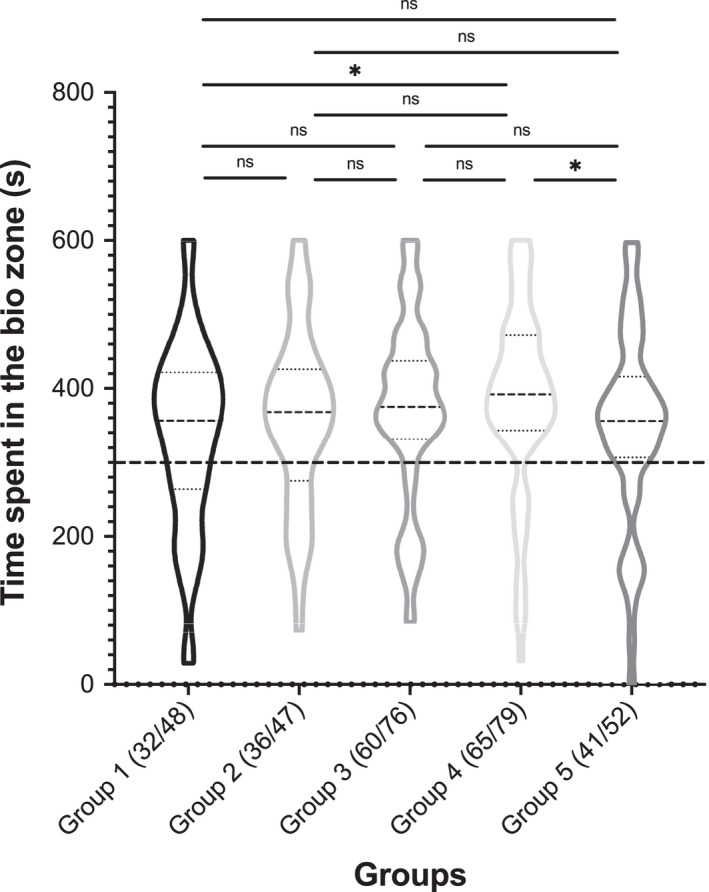
Time spent in the biozone in M‐POC experiment. The number of bats in each group spending more than half of total time in the biozone is indicated by fractional numbers. The distance between the two walls of the balloons represents number of bats. The thick dashed line denotes half (300 s) of total observation time (600 s). The thick dashed line in the balloons indicates the mean of data from all individual bats in a group, and the thin dashed lines above and below it represent 3 quarters and one quarter of total time spent in the biozone, respectively. ns: not significant. *p < .05

Results showed that mother bats spent an average of 365.63 s in the biozone and 226.51 s in the alien zone. Among these bats, 234 mother bats (77.48%) spent more than half of total time in the biozone, while 68 mother bats (22.52%) spent more than half of total time in the alien zone. Results also showed that the mother bats in all five age groups spent more time in the biozone than in the alien zone (Group 1: *p* < .05, Group 2: *p* < .01, Group 3: *p* < .01, Group 4: *p* < .01, and Group 5: *p* < .01). The amount of time that mother bats spent in the biozone was slightly increased over the age of pups, except those in Group 5 (Figure 3). Pairwise comparison revealed a significant difference in time spent in the biozone between Group1 and Group 4 (Wilcoxon test: *p* = .029) and between Group 4 and Group 5 (Wilcoxon test: *p* = .046) with mother bats in Group 4 spending more time in the biozone (Figure 3).

### 3.2 Groupmate odorant choice (GOC) experiment

**FIGURE 4:**
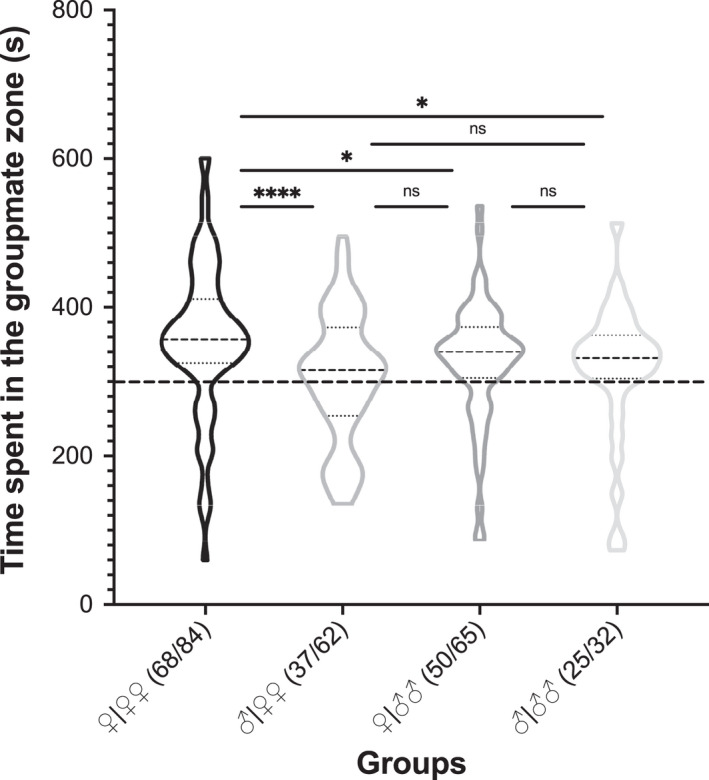
Time spent in the groupmate zone in GOC experiment. The number of bats in each group spending more than half of total time in the groupmate zone is indicated by fractional numbers. The distance between the two walls of the balloons represents number of bats. The thick dashed line denotes half (300 s) of total observation time (600 s). The thick dashed line in the balloons indicates the mean of data from all individual bats in a group, and the thin dashed lines above and below it represent 3 quarters and one quarter of total time spent in the biozone, respectively. ns: not significant. *p < .05. **p < .01

Results showed that bats spent an average of 330.89 s in the groupmate zone and 269.52 s in the nongroupmate zone. In total, 180 of 243 (74.07%) bats spent more than half of total time in the groupmate zone, while 63 (25.93%) bats spent more than half of total time in the nongroupmate zone. However, male bats in Group ♂|♀♀ showed no significant difference in preference to scent from female groupmates and nongroupmates (*p* > .05). Pairwise comparison between groups ♀|♀♀ and ♂|♀♀, between groups ♀|♀♀ and ♀|♂♂, and between groups♀|♀♀ and ♂|♂♂ revealed that bats in Group ♀|♀♀ spent more time in the groupmate zone than those in other groups (Figure 4).

### 3.3 Sex odorant choice (SOC) experiment

**FIGURE 5:**
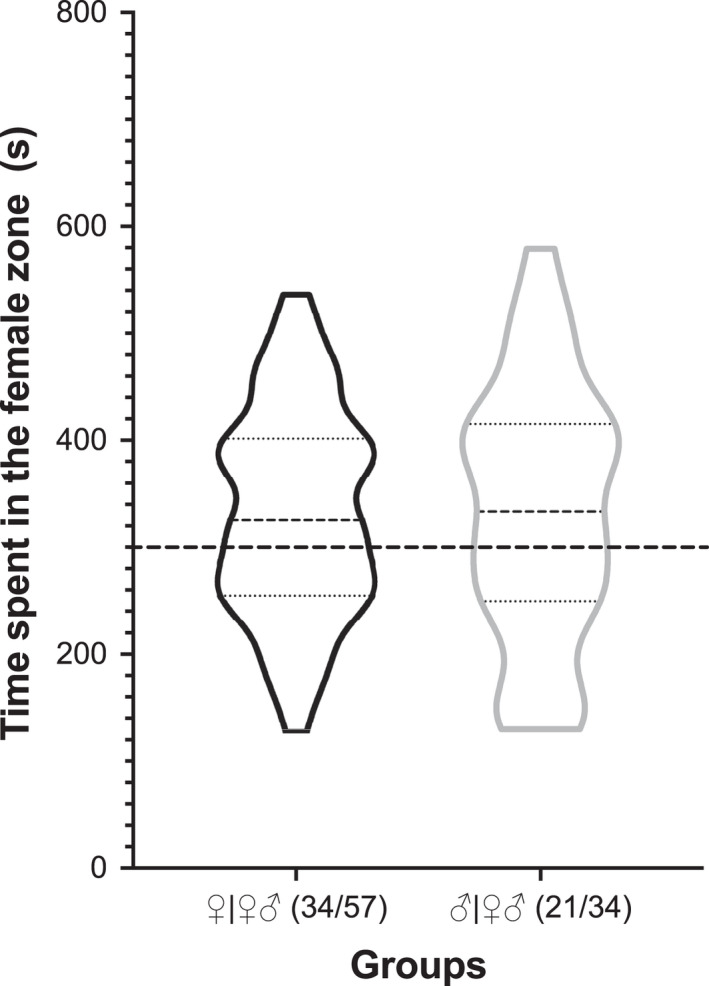
Time spent in the female zone in SOC experiment. The number of bats in each group spending more than half of total time in the female zone is indicated by fractional numbers. The distance between the two walls of the balloons represents number of bats. The thick dashed line denotes half (300 s) of total observation time (600 s). The thick dashed line in the balloons indicates the mean of data from all individual bats in a group, and the thin dashed lines above and below it represent 3 quarters and one quarter of total time spent in the biozone, respectively

Results showed that bats spent an average of 332.70 s in the female zone and 267.30 s in the male zone. In total, 55 (60.44%) bats spent more than half of total time in the female zone, and 36 (39.56%) bats spent more than half of total time in the male zone. Unexpectedly, both female and male bats were found to have no difference in preference to scent from the same or opposite sex of bats of the same group (*p* > .05) (Figure 5).

## 4 DISCUSSION

Results of the mother–pup odorant choice **(**M‐POC) experiment suggest that mother bats can recognize their own pups by scent as expected. Effective mother–pup recognition has been shown to avoid feeding alien offspring (commonly referred to as allo‐suckling) and protect pups from being killed by predators (Bohn et al., 2009). Mother–offspring olfactory recognition has been widely investigated and is known to be ubiquitous in colonial mammals, including seal (Wierucka et al., 2018), seal lion (Pitcher et al.2011), mouse (Logan et al.2012), and bat (Gustin & McCracken, 1987; Watkins & Shump, 1981). Because the age of pups may affect results, we tested mother bats according to the ages of their pups. The observation that mother bats spent more time in the biozone with scent from older pups suggests that the composition of scent from pup changes over time. It is known that the chemical composition of body odors changes in an age‐dependent manner in a variety of animals, such as mouse (Osada et al., 2003), meadow vole (Ferkin, 1999), black‐tailed deer (Muller‐Schwarze, 1971), rabbit (Goodrich & Mykytowycz, 1972), and human being (Mitro et al., 2012).

In the groupmate odorant choice (GOC) experiment, except for bats in Group ♂|♀♀, bats in other groups were more attracted to the scent from their groupmates regardless of sex (Figure 4). This finding is consistent with that of a previous study showing that bats often exhibit a group scent profile which can be recognized by other groupmates (Bloss, 1999). The recognition of groupmates allows philopatric colonial bat species to form stable associations within closed colonies or harems (Bloss et al., 2002; Kerth et al., 2000; Kerth, Mayer, et al., 2002; Kerth, Safi, et al., 2002; Veith et al., 2004; Voight & von Helverson, 1999). An exception was found in Group ♂|♀♀, in which male bats showed no significant difference in preference to scent from female groupmates and nongroupmates. This situation may be related to the characteristics of *Tylonycteris pachypus* that males tend to be solitarily, while females are gregarious (Zhang et al., 2004). As territory is a valuable resource for males (Caspers & Voigt, 2009), scent making is considered as a way of advertising ownership of a territory (Gosling & Roberts, 2001). For instance, males of greater sac‐winged *Saccopteryx bilineata* bats use scent to mark their territory against foreign male but not female competitors (Caspers & Voigt, 2009).

Female bat philopatry has been found in some bat species, such as *Macroderma gigas* (Worthington et al., 1994), *Rhinolophus ferrumequinum* (Rossiter et al., 2001), and *Myotis bechsteinii* (Kerth, Mayer, et al., 2002; Kerth, Safi, et al., 2002). Female groupmates are known to be very cooperative as evidenced by the presence of nonreproductive females in groups with pregnant or lactating bates (Kerth & König, 1999; Kerth et al., 2001). As our experiment was performed in artificial environments, how female *T*. *pachypus* bats live together peacefully in wildlife remains to be investigated.

Results of the sex odorant choice (SOC) experiment showed that both females and males had no difference in preference to the scent from bats of the same or opposite sex in the same group (Figure 5). As with other mammals, male bat offspring rarely mate within their natal group as they are usually chased away by the dominant male (Dobson, 1982; Greenwood, 1980; Moore & Ali, 1984), thus reducing inbreeding and increasing genetic diversity and survival advantage (Zhang et al., 2011). Another reason for this observation is that our sex choice experiment was performed in nonmating season, July to September, in which male bats (*Saccopteryx bilineata*) have been shown to lack male‐specific scent (Caspers et al., 2011). Another possible reason is that male bats in the same group might be siblings or father and son with a strong kinship so that they were more attracted to each other.

In conclusion, we found that female *T*. *pachypus* bats recognized their pups by scent. We also found that both female and male *T*. *pachypus* bats recognized their groupmates by scent and that female bats were only attracted to the scent from their groupmates. Our observation also showed that male bats were much more attracted to the scent from male groupmates than that from female groupmates. In addition, we found that both female and male bats had no preference to the scent from the same or opposite‐sex groupmates. As this result is unexpected, further studies are warranted to investigate the conditions and life periods in which bats are interested in the scent from their opposite‐sex groupmates. The mechanisms by which female bat groupmates are more attracted to each other to live together in wildlife also remain to be investigated.

## CONFLICT OF INTEREST

None declared.

## AUTHOR CONTRIBUTIONS

**Libiao Zhang:** Conceptualization (equal); funding acquisition (equal); methodology (equal); writing–review and editing (equal). **Jie Liang:** Formal analysis (equal); methodology (equal); software (equal); writing–original draft (equal). **Jian Yang:** Conceptualization (equal); investigation (equal); methodology (equal); resources (equal). **Yi Chen:** Investigation (equal); methodology (equal); resources (equal).

## ETHICAL APPROVAL

Collection of bats was done according to the guidelines of Regulations for the Administration of Laboratory Animals (Decree No. 2, State Science and Technology Commission, People's Republic of China). The animal experiments performed in this study were approved by the Guangdong Entomological Institute Administrative Panel on Laboratory Animal Care (No. GDEI‐AE‐2006001).

## Data Availability

Data generated in this study have been deposited in the Dryad Digital Repository (https://doi.org/10.5061/dryad.dncjsxkwv).
